# Splenic enlargement induced by preoperative chemotherapy is a useful indicator for predicting liver regeneration after resection for colorectal liver metastases

**DOI:** 10.1186/s12957-020-01918-4

**Published:** 2020-06-23

**Authors:** Takanori Konishi, Hiroyuki Yoshidome, Hiroaki Shimizu, Hideyuki Yoshitomi, Katsunori Furukawa, Tsukasa Takayashiki, Satoshi Kuboki, Shigetsugu Takano, Masaru Miyazaki, Masayuki Ohtsuka

**Affiliations:** 1 Department of General Surgery, Chiba University Graduate School of Medicine, 1-8-1 Inohana, Chuo-Ku, Chiba, 260-8670 Japan; 2 Department of Surgery, Oami Municipal Hospital, 884-1 Tomida, Oami-Shirasato-shi, Chiba, 299-3221 Japan; 3 Surgery and Digestive Disease Center, International University of Health and Welfare, Mita Hospital, 1-4-3 Mita, Minato-Ku, Tokyo, 108-8329 Japan

**Keywords:** Hepatectomy, Splenomegaly, Conversion chemotherapy, Colorectal liver metastases, Liver regeneration

## Abstract

**Background:**

Conversion chemotherapy may downsize unresectable colorectal liver metastases (CRLMs), but may cause liver injury and splenic enlargement. The effect of preoperative chemotherapy on liver regeneration after liver resection remains undetermined. The aim of this study was to examine whether splenic enlargement induced by preoperative chemotherapy is an indicator to identify high-risk patients for impaired liver regeneration and liver dysfunction after resection.

**Methods:**

We retrospectively reviewed 118 Japanese patients with CRLMs. Fifty-one patients had conversion chemotherapy. The other 67 patients underwent up-front liver resection. We clarified effects of conversion chemotherapy on splenic volume, liver function, and postoperative liver regeneration. Perioperative outcome was also analyzed.

**Results:**

A ratio of the splenic volume before and after chemotherapy (SP index) in the oxaliplatin-based chemotherapy group was significantly greater than other chemotherapy groups after 9 or more chemotherapy cycles. Patients whose SP index was 1.2 or more had significantly higher indocyanine green retention rate at 15 min (ICG-R15) than patients without chemotherapy. Analyses of covariance showed liver regeneration rate after resection was decreased in patients whose SP index was 1.2 or more. The incidence of postoperative liver dysfunction in patients whose SP index was 1.2 or more was significantly greater than patients without chemotherapy. Multivariate analysis showed SP index was a significant predictive factor of impaired liver regeneration.

**Conclusions:**

Splenic enlargement induced by preoperative chemotherapy was a useful indicator for impaired liver regeneration after resection and a decision-making tool of treatment strategy for unresectable CRLMs.

## Background

Hepatic resection is a possibly curative treatment for colorectal liver metastases (CRLMs). However, up to 80% of CRLMs are initially diagnosed as unresectable [1]. Currently, 5-fluorouracil and folinic acid in combination with either oxaliplatin or irinotecan are commonly used cytotoxic regimens for colorectal cancer [2]. Biologics such as anti-VEGF and anti-EGFR monoclonal antibody are often used simultaneously for patients with unresectable colorectal cancer, as they improve the response and resection rate [3–5]. Thus, recent advances in chemotherapy for colorectal cancer may render unresectable CRLMs amenable to resection. Adam et al. demonstrated that conversion chemotherapy may downsize unresectable CRLMs [6]. They proceeded to hepatic resection in 12.5% of patients with initially unresectable CRLM, with a 5-year survival rate after liver resection of 33%. However, preoperative chemotherapy causes liver injury, such as sinusoidal obstructive syndrome or steatohepatitis, which may affect postoperative outcomes after liver resection [7]. Sinusoidal obstruction syndrome associated with oxaliplatin is reported to cause an increased risk of postoperative morbidity and long hospital stay in patients undergoing major hepatectomy [8]. Sinusoidal damage is likely to elevate portal venous pressure, which may cause enlargement of the spleen. Therefore, an increase in the splenic volume is reported to be a predictive marker of the development of sinusoidal obstruction syndrome [9].

Liver regeneration is an important phenomenon to maintain liver volume and function after loss of liver parenchyma, such as with liver transplantation and hepatic resection. Chronic hepatitis and cirrhosis are associated with poor postoperative liver regeneration [10], and insufficient regeneration after major hepatectomy is likely to lead to liver failure [11]. Although chemotherapy is sometimes used before hepatectomy for CRLMs, the association between preoperative chemotherapy and liver regeneration after hepatic resection has not been well defined. It may be important to identify patients who will have insufficient liver regeneration and postoperative liver failure before operation. We hypothesized that preoperative chemotherapy may increase splenic volume, which might be associated with compromised liver regeneration. The objective of this study was to investigate splenic enlargement induced by preoperative chemotherapy for CRLMs as a marker for selecting the patients with impaired liver regeneration after liver resection.

## Methods

### Patients and preoperative evaluation

We retrospectively analyzed the medical records of 118 Japanese patients with resectable, not optimally resectable, or initially unresectable CRLM seen at the Department of General Surgery at Chiba University (Chiba, Japan) between 2005 and 2013. All patients were evaluated preoperatively with abdominal ultrasonography, thoracoabdominal dynamic multidetector-row computed tomography (MD-CT), and magnetic resonance imaging. The remnant liver functional reserve was predicted from the indocyanine green retention rate at 15 min. The future remnant liver volume was predicted by CT volumetry in every patient for whom liver resection was planned. Patients with a predicted remnant liver volume ≥ 35% of the total liver volume underwent liver resection without preoperative portal vein embolization or staged hepatectomy. The resectability of each patient was evaluated based on modification of the previous report [12]. Briefly, patients whose predicted remnant liver volume is far less than 35% of the total liver because of biloblar multiple tumors or the invasion or close to all of the hepatic veins or both portal branches were defined as initially unresectable CRLM. Patients who have poor prognostic factors such as extrahepatic metastases were defined as not optimally resectable CRLM.

Of the 118 patients analyzed, 51 received conversion chemotherapy for not optimally resectable or initially unresectable CRLMs. Chemotherapy regimen was chosen based on the guideline from Japanese Society for cancer of the Colon and Rectum. Second-line regimen chemotherapy was performed when first-line regimen was not effective. Hepatic resection was proceeded when unresectable CRLMs were converted to technically resectable by conversion chemotherapy. The other 67 patients underwent liver resection without preoperative chemotherapy during the same period. All patients have given informed consent in accordance with the ethical standards. The study protocol has been approved by the institute’s committee on human research. The approval code obtained from the corresponding ethical committee on human research was #3150 (Chiba University School of Medicine). We retrospectively analyzed details of the metastatic tumors, operative data, and postoperative complications as defined by the Clavien–Dindo classification [13]. Steatosis was diagnosed when liver attenuation was less than or equal to spleen attenuation minus 10 HU in unenhanced CT according to the previous report [14].

### Splenic volume and liver volume analysis

For patients who received chemotherapy, an enhanced MD-CT was performed before and after chemotherapy in order to assess the indications for hepatic resection. The splenic volume before (pre-SP) and after chemotherapy (post-SP) was measured in an identical manner by MD-CT. A change in the splenic volume during chemotherapy (SP index) was calculated as the ratio of post-SP to pre-SP. The preoperative entire liver volume (ELV) exclusive of CRLMs and the preoperative future remnant liver volume (FRLV) were measured by MD-CT. The ratio of FRLV to ELV was calculated as 100 × FRLV/ELV. CT was performed in patients 7 days after liver resection to assess postoperative complications and calculate the remnant liver volume (RLV_7_). The liver regeneration rate was defined as RLV_7_/FRLV. Splenic and liver volumes were measured by SYNAPSE VINCENT (Fuji Photo Film CO., Ltd. Japan).

### Liver regeneration analysis

In order to identify whether chemotherapy-induced liver damage affected liver regeneration after hepatectomy, the cutoff value of the SP index was defined by the receiver-operating characteristic (ROC) curve. According to this value, we divided the patients into three groups. Characteristics of the patients, operative data, liver regeneration rate, and postoperative course were analyzed in these three groups.

### Statistical analysis

Summary statistics were constructed for baseline values, employing frequencies and percentages for categorical data and means and standard deviations for continuous variables. We compared patient characteristics using Fisher’s exact test for categorical outcomes, Mann-Whitney *U* test for continuous unpaired outcomes, and Wilcoxon signed-rank test for continuous paired outcomes. We compared the patients’ data among the three groups by using the chi-square test for categorical outcomes and the Kruskal-Wallis test for continuous outcomes. The relationship between a change in splenic volume and liver regeneration was assessed by using analysis of covariance to adjust for imbalances in the ratio of FRLV to ELV. Multivariate analysis was performed using the logistic regression model. A *p* value of < 0.05 (two-tailed tests) was considered to be significant. All statistical analyses were performed using the SPSS 18.0 software program (Chicago, IL).

## Results

### Patients and chemotherapy

Among the 51 patients who received chemotherapy, 43 were able to proceed to hepatectomy, while 8 still had unresectable CRLMs after chemotherapy. The other 67 patients underwent up-front hepatectomy. The numbers of liver metastases, patients with positive lymph node metastases of the primary lesion, and patients with extrahepatic metastases in those treated with chemotherapy were significantly greater than in those without chemotherapy. Of those receiving chemotherapy, 45 patients had a first-line regimen and 6 had a second-line regimen. Oxaliplatin-based chemotherapy was administered in 29 patients and irinotecan-based therapy in 20 patients. Fluorouracil plus leucovorin was given to 2 patients. The median chemotherapy course was 9 cycles, and 27 patients received 9 cycles or more.

### Changes in splenic volume

**Fig 1:**
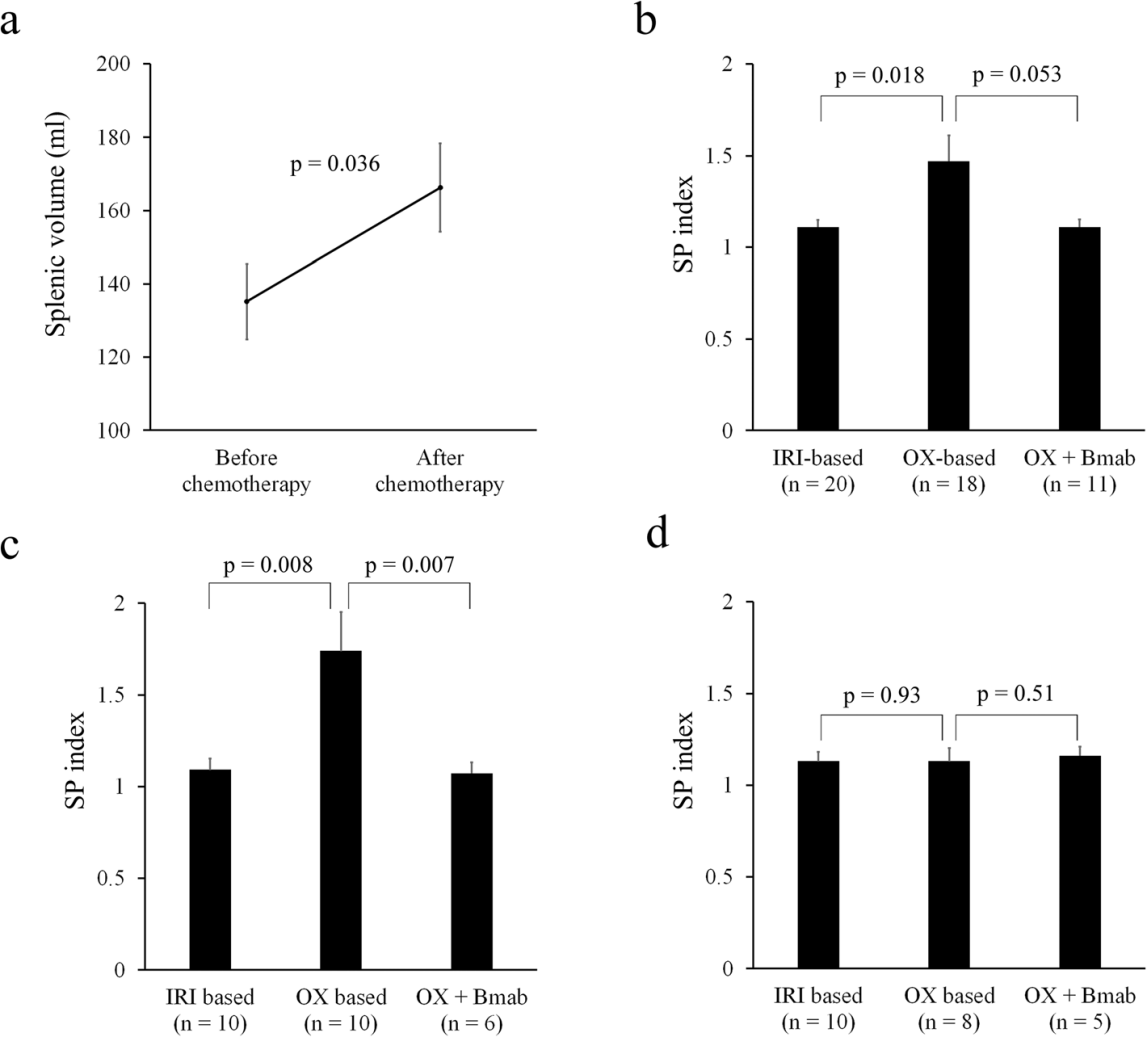
Changes in splenic volume during chemotherapy. **a** Splenic volume before and after preoperative chemotherapy (*n* = 51). **b** The relationship between SP index and chemotherapeutic regimen. IRI-based: FOLFIRI/IRIS with or without biologics (*n* = 20). OX-based: FOLFOX/CapeOX with or without anti-EGFR monoclonal antibodies (*n* = 18). OX + Bmab: FOLFOX/CapeOX with bevacizumab (*n* = 11). **c** SP index in patients who had 9 or more cycles of chemotherapy. **d** SP index in patients who had 8 or fewer cycles of chemotherapy. Data are mean ± standard error of the mean. A comparison was performed using the Mann-Whitney *U* test. A *p* value < 0.05 was considered to be significant

**Fig 2:**
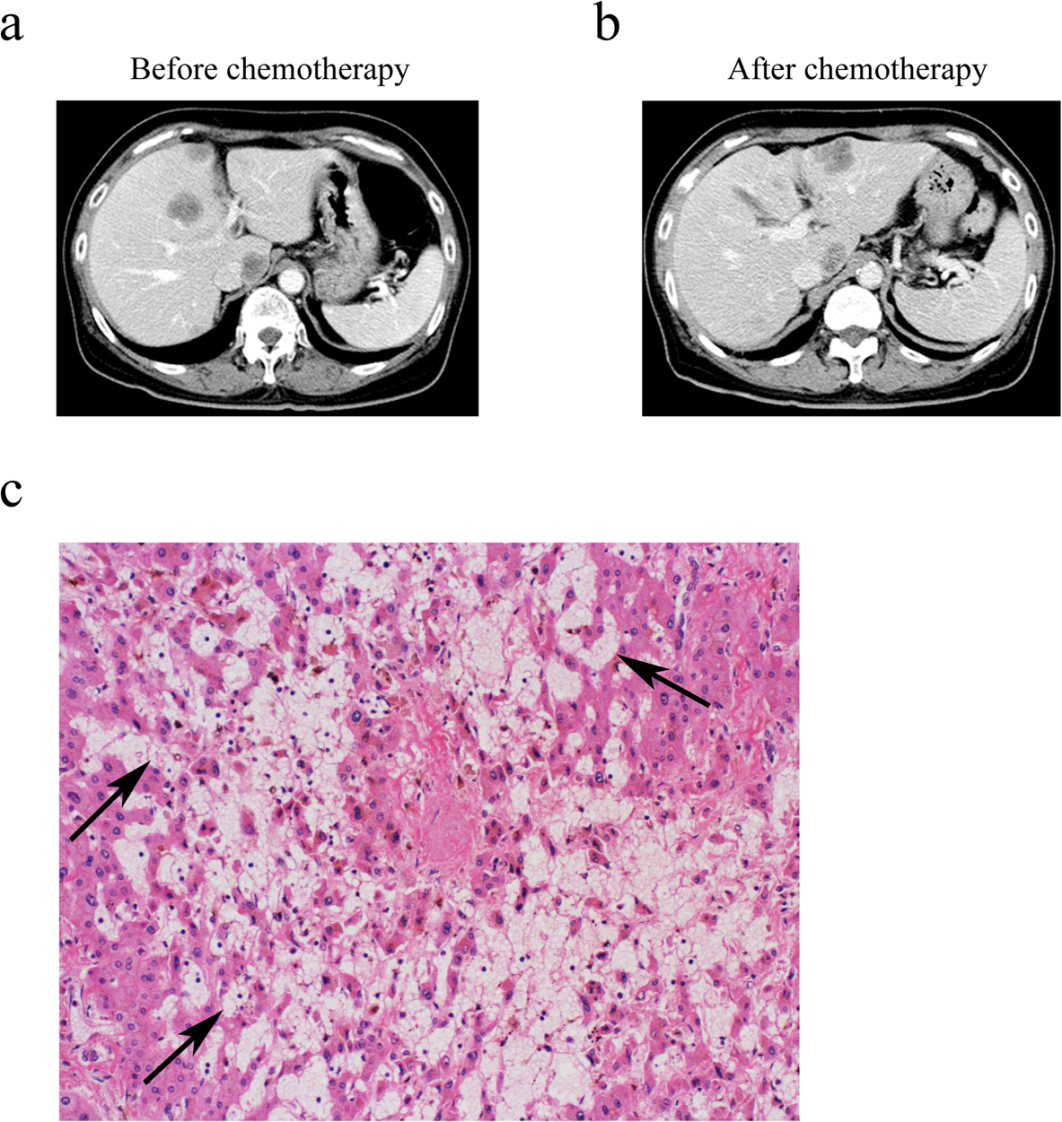
Representative case of splenomegaly induced by chemotherapy. **a** Abdominal dynamic multidetector-row computed tomography (MD-CT) findings before chemotherapy. **b** MD-CT findings after 20 cycles of oxaliplatin-based chemotherapy. The splenic volume increased during chemotherapy (SP index = 1.34). **c** Histological analysis (hematoxylin and eosin staining) of the liver after resection. The non-tumoral liver developed sinusoidal obstruction syndrome. Arrows indicate dilatation of sinusoids. Original magnification is × 200

To determine whether chemotherapy affects splenic volume, volumetric analyses of the spleen before and after chemotherapy were performed. The splenic volume significantly increased after chemotherapy (Fig. 1a, *p* = 0.036). To assess whether splenic volume was affected by specific chemotherapy regimens, we divided patients into three groups stratified by regimen: FOLFIRI/IRIS with or without targeted therapies (IRI-based, *n* = 20), FOLFOX/CapeOX with or without anti-EGFR monoclonal antibodies (OX-based, *n* = 18), and FOLFOX/CapeOX with bevacizumab (OX-based + Bmab, *n* = 11). The two patients who received fluorouracil + leucovorin were excluded in this analysis. The SP index in the OX-based group was significantly greater than that of the IRI-based group (Fig. 1b, *p* = 0.018). Among patients who underwent 9 or more chemotherapy cycles, the SP index of the OX-based group was significantly greater than that of IRI-based and OX-based + Bmab groups (Fig. 1c, *p* = 0.008, 0.007, respectively). There was no significant difference among patients receiving 8 or fewer cycles of chemotherapy (Fig. 1d). Here, we show a representative case of chemotherapy-induced splenomegaly. A patient underwent 20 cycles of OX-based chemotherapy before liver resection. The splenic volume increased during the chemotherapy (Fig. 2a; SP index = 1.34), and the histological analysis showed severe sinusoidal obstruction syndrome in the resected liver specimen after resection (Fig. 2b).

### Liver regeneration and postoperative course after hepatectomy

**Fig 3:**
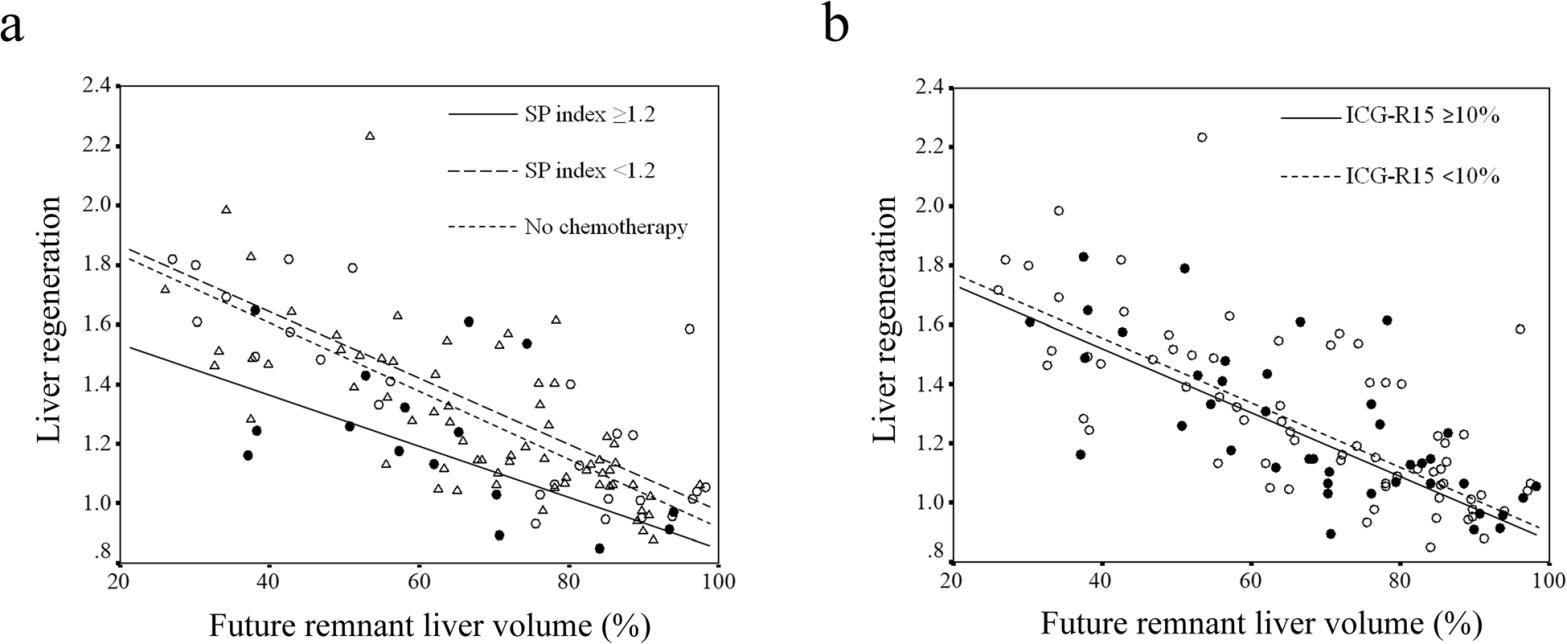
Relationship between splenic enlargement and liver regeneration. **a** The relationship between splenic enlargement and liver regeneration. SP index ≥ 1.2: changes in splenic volume during chemotherapy were 1.2 or more (●, *n* = 16). SP index < 1.2: changes in splenic volume during chemotherapy were less than 1.2 (○, *n* = 27). No chemotherapy: patients received no preoperative chemotherapy before hepatectomy (▵, *n* = 67). There was statistical significance among these groups (SP index ≥ 1.2 vs SP index < 1.2 or no chemotherapy; *p* = 0.021, 0.033, respectively) defined by analyses of covariance. **b** The relationship between ICG-R15 and liver regeneration. ICG-R15 ≥ 10% (●, *n* = 40), ICG-R15 < 10% (○, *n* = 70). There was no significant difference in liver regeneration between the two groups (*p* = 0.40)

To determine whether preoperative chemotherapy-induced splenomegaly may affect liver regeneration after hepatectomy, we defined the cutoff value of the SP index by the ROC curve as 1.2. We divided the patients who underwent hepatectomy into three groups: those receiving chemotherapy and whose SP index was 1.2 or more (*n* = 16), those receiving chemotherapy and whose SP index was less than 1.2 (*n* = 27), and those without preoperative chemotherapy (*n* = 67). Table 1 shows patient characteristics in each group. There were no significant differences in sex, age, body mass index (BMI), prevalence of diabetes mellitus, hepatic virus infection, or steatosis in the three groups. The ICG-R15 in patients whose SP index was 1.2 or more was significantly greater than in patients without preoperative chemotherapy. Analyses of covariance showed that the liver regeneration rate in patients whose SP index was 1.2 or more was significantly lower than in patients whose SP index was less than 1.2 and in patients without preoperative chemotherapy, adjusting for imbalances of the ratio of FLRV to ELV (Fig. 3a, *p* = 0.021, 0.033, respectively), but ICG-R15 did not significantly affect liver regeneration in this set of the patients (Fig. 3b, *p* = 0.40).

**Table 1:** Characteristics of patients undergoing liver resection for colorectal liver metastases

Characteristics	Chemotherapy		No chemotherapy (*n* = 67)	*p* value
	SP index ≥ 1.2 (*n* = 16)	SP index < 1.2 (*n* = 27)		
Gender (male/female)	11/5	15/12	35/32	0.49
Age	57.8 ± 13.3	62.1 ± 10.3	64.4 ± 9.5	0.10
BMI	23.5 ± 3.6	21.5 ± 2.9	22.8 ± 3.9	0.14
Diabetes mellitus (+)	4	5	8	0.38
Hepatitis virus (+)	0	0	2	0.52
Number of chemotherapy cycles (median, range)	12 (5–27)	8 (1–43)	–	0.27
ICG-R15 (%)	13.5 ± 9.5	9.4 ± 4.8	8.6 ± 4.8	0.036
Steatosis before chemotherapy	1	1	–	1.00
Steatosis before operation	3	3	3	0.14

Continuous data are presented as mean ± standard deviation

*BMI* body mass index, *ICG-R15* indocyanin green retention rate at 15 min

To identify the factors that affect liver regeneration after hepatectomy and to determine whether changes in splenic volume influence the operative and postoperative course, we selected a subset of patients undergoing major hepatectomy (resection of three or more segments in Couinaud’s classification) (*n* = 56). The patients’ characteristics are shown in Table 2. The splenic volume and levels of serum aspartate and alanine aminotransferase before hepatectomy in patients whose SP index was 1.2 or more (*n* = 12) were significantly greater than in those whose SP index was less than 1.2 (*n* = 14) and in patients without preoperative chemotherapy (*n* = 30). Platelet counts before hepatectomy in patients whose SP index was 1.2 or more were significantly lower than in patients without chemotherapy and serum albumin levels before hepatectomy in patients whose SP index was 1.2 or more were significantly greater than in patients without chemotherapy. We analyzed factors affecting liver regeneration after hepatectomy with logistic and multiple linear regression, adjusting for possible differences in baseline scores and background characteristics such as sex, age (more than 70 years old), BMI (more than 25), diabetes mellitus, number of chemotherapy cycles (9 cycles or more), SP index (1.2 or more), platelet counts before operation (more than the lower limit of normal), and concomitant portal vein embolization. The SP index was a significant factor predictive of impaired liver regeneration on multivariate analysis (odds ratio, 7.889; 95% CI, 1.132 ~ 54.961; *p* = 0.037).

**Table 2:** Characteristics and preoperative data of patients undergoing major hepatectomy

Characteristics	Chemotherapy		No chemotherapy (*n* = 30)	*p* value
	SP index ≥ 1.2 (*n* = 12)	SP index < 1.2 (*n* = 14)		
Gender (male/female)	8/4	8/6	14/16	0.48
Age	58.3 ± 12.4	63.6 ± 10.3	63.0 ± 10.0	0.38
BMI	22.9 ± 3.1	21.3 ± 2.7	22.8 ± 3.8	0.23
Diabetes mellitus (+)	4	3	5	0.49
Hepatitis virus (+)	0	0	2	0.41
Number of chemotherapy cycles (median, range)	12 (5–27)	8 (1–23)	–	0.21
SP index	1.65 ± 0.60	1.04 ± 0.05	–	< 0.001
Splenic volume before chemotherapy	131.7 ± 48.2	112.4 ± 49.4	–	0.23
Splenic volume before hepatectomy	214.3 ± 88.8	117.7 ± 56.9	160.9 ± 162.0	0.004
Laboratory tests before hepatectomy				
Platelet count (× 10^9^/L)	171 ± 60	231 ± 97	234 ± 72	0.029
AST (IU/L)	33.3 ± 11.7	24.8 ± 7.8	26.7 ± 9.3	0.035
ALT (IU/L)	30.5 ± 18.9	19.2 ± 13.6	22.2 ± 13.5	0.032
ALP (IU/L)	371.6 ± 161.3	410.9 ± 186.3	303.8 ± 159.2	0.050
Albumin (g/dL)	3.9 ± 0.3	3.9 ± 0.4	4.1 ± 0.2	0.014
T-Bil (mg/dL)	0.9 ± 0.4	0.9 ± 0.4	0.9 ± 0.3	0.94
PT-INR	1.02 ± 0.08	1.04 ± 0.09	1.08 ± 0.10	0.17
ICG-R15 (%)	12.3 ± 5.9	10.2 ± 6.0	7.9 ± 3.9	0.061
Future remnant liver volume (%)	55.9 ± 12.8	49.3 ± 18.5	53.5 ± 13.8	0.42
TSH/PVE	3	4	2	0.12

Continuous data are presented as mean ± standard deviation

*BMI* body mass index, *AST* aspartate aminotransferase, *ALT* alanine aminotransferase, *ALP* alkaline phosphatase, *T-Bil* total bilirubin, *PT-INR* prothrombin time international normalized ratio, *ICG-R15* indocyanine green retention rate at 15 min, *TSH* two stage hepatectomy, *PVE* portal vein embolization

Surgical data and postoperative events are shown in Table 3. Operation time and blood loss during surgery in patients whose SP index was 1.2 or more were significantly greater than in patients without preoperative chemotherapy. We investigated the incidence of postoperative liver failure in the three groups. This was defined as hyperbilirubinemia (at least twice the upper limit of normal) on or after postoperative day 5, according to the classification of the International Study Group of Liver Surgery [15]. The incidence of postoperative liver failure in patients whose SP index was 1.2 or more was significantly greater than in patients without chemotherapy (Table 3). On the other hand, there was no significant difference between patients whose SP index was less than 1.2 and those without chemotherapy.

**Table 3:** Operative and postoperative outcome after major hepatectomy

	Chemotherapy		No chemotherapy (*n* = 30)	*p* value
	SP index ≥ 1.2 (*n* = 12)	SP index < 1.2 (*n* = 14)		
Operation time (min)	388 (255–551)	306 (260–739)	289 (190–430)	0.035
Blood loss (ml)	1368 (225–3420)	661 (335–2560)	570 (285–4935)	0.024
Intraoperative transfusion	6 (50%)	7 (50%)	7 (23.3%)	0.12
Postoperative hyperbilirubinemia	6 (50%)	4 (28.6%)	4 (13.3%)	0.043
Morbidity (Clavien–Dindo classification III≦)	2 (16.7%)	3 (21.4%)	3 (10%)	0.58
Biliary leakage	2	2	3	
Intra-abdominal abscess	1	2	1	
Mortality	0	0	0	

Continuous data are presented as median and range

## Discussion

Recent advances in chemotherapy for colorectal cancer have improved the resection rate and prolonged survival for patients with initially unresectable CRLMs [1, 16–18]. Although several advantages of preoperative chemotherapy for CRLMs have been demonstrated, drawbacks have also been recognized [19, 20]. Cytotoxic agents cause certain types of hepatic parenchymal injury, such as steatohepatitis or sinusoidal obstruction syndrome [7, 21], but the actual effect of chemotherapy-induced hepatotoxicity on liver regeneration after resection and postoperative complications remains unclear. This study revealed that an increase in splenic volume mediated by preoperative chemotherapy is associated with poor preoperative liver function, which is consistent with the previous report that showed oxaliplatin-based chemotherapy increases splenic volume as a result of sinusoidal injury [9]. Furthermore, we clarified that patients with splenic enlargement during chemotherapy had impaired liver regeneration and liver dysfunction after liver resection.

Long-term chemotherapy reportedly fails to improve the response rate, but it increases the incidence of sinusoidal injury, thus leading to postoperative liver failure [22]. In the current study, among patients undergoing 9 or more cycles of chemotherapy, splenomegaly in the OX-based group was significantly greater than that in the IRI-based and OX-based + Bmab-treated groups, which suggests that long-term oxaliplatin-based chemotherapy favorably causes splenic enlargement. Consistent with previous reports [23, 24], the current study also demonstrated that bevacizumab is likely to inhibit enlargement of the spleen, probably because of a decrease in sinusoidal injury. Patients with splenic enlargement had increased levels of aspartate aminotransferase and alanine aminotransferase and worse ICG-R15 those of which relate to liver injury and deterioration of hepatic function. Taken together, the evidence suggests that changes in splenic volume, which can be easily assessed before hepatectomy without invasive examination, are likely to be a marker of chemotherapy-induced liver damage and a decision-making marker of selecting chemotherapy regimen for conversion surgery.

The influence of preoperative chemotherapy on liver regeneration after liver resection is still controversial. Although some reports showed that preoperative chemotherapy itself does not affect liver regeneration and postoperative course [25–27], few studies have argued the adverse effects of chemotherapy-induced hepatotoxicity on early liver regeneration after liver resection. Current study suggested that splenic enlargement which represents liver injury caused by chemotherapy is related to impaired liver regeneration after hepatectomy. Our results also showed that there was no significant difference in liver regeneration between patients whose SP index was less than 1.2 and patients without preoperative chemotherapy. By measuring splenic volume during preoperative chemotherapy, we may preoperatively select the patients who will suffer chemotherapy-mediated impaired liver regeneration after resection.

Liver regeneration is achieved through multiple signaling pathways by mitogenic growth factors, such as HGF, EGF, and TGF-α, leading eventually to hepatocyte growth and proliferation [28]. Sinusoidal endothelial cells play an important role in the release of HGF through VEGFR2 [29]. We measured liver volume 7 days after hepatectomy to assess early liver regeneration, which may directly correlate with postoperative liver failure. In the current study, patients with an SP index ≥ 1.2 had less early liver regeneration after adjusting for imbalances of the FRLV, but there was no correlation between early liver regeneration and ICG-R15 as previously reported [30]. These results suggested that splenic enlargement is an indicator of liver regeneration after resection, while ICG-R15 represents hepatic functional reserve. Splenic volume is negatively correlated with liver regeneration after major hepatectomy [31]. Liver regeneration may be inhibited by splenic upregulation of TGF-β1 and downregulation of HGF [32, 33]. These findings suggest that a prolonged duration of OX-based chemotherapy that induces splenomegaly may affect liver regeneration. Furthermore, the sinusoidal obstruction syndrome model induced by monocrotaline resulted in decreased mRNA expression of mitogenic growth factors and impaired hepatic regeneration in rats [34]. Sinusoidal obstruction syndrome is likely to inhibit liver hypertrophy after first hepatectomy with portal vein embolization in a two-stage hepatectomy [35]. It seems likely that splenic enlargement induced by long-term preoperative chemotherapy influences liver regeneration after resection for CRLMs. Consistent with these findings and based on our multivariate analysis, an SP index of ≥ 1.2 is a possible candidate marker for predicting impaired liver regeneration.

A previous study showed that sinusoidal injury increased the risk of greater morbidity and longer hospital stay after major hepatectomy [8]. An increase in splenic volume has been correlated with major complications after hepatic resection [36]. Current study showed that splenic enlargement was associated with a longer operative time and increased blood loss during operation. Furthermore, patients with an SP index ≥ 1.2 were likely to be at greater risk of postoperative liver failure as defined by hyperbilirubinemia, suggesting that preoperative chemotherapy-induced splenic enlargement may be a caution for the occurrence of postoperative liver failure after major liver resection. The limitations of this study are the number of patients and lack of the evaluation of liver function after liver resection. Although previous report showed the discrepancy of the volume and the function of remnant liver [37], the results of the current study may be helpful for considering continuation of chemotherapy or operative risk after conversion surgery to avoid occurrence of liver dysfunction after hepatectomy.

## Conclusions

Long-term oxaliplatin-based chemotherapy caused an increase in splenic enlargement. The splenic enlargement may cause impaired liver regeneration after hepatectomy and is likely to be related to occurrence of postoperative liver failure. The ratio of splenic volume before and after chemotherapy may be a useful indicator for decision-making of treatment strategy for unresectable CRLMs.

## Data Availability

The datasets used for this study are available from corresponding author on reasonable request.

## Abbreviations

CRLM: Colorectal liver metastases
VEGF: Vascular endothelial growth factor
MD-CT: Multidetector computed tomography
ELV: Entire liver volume
FRLV: Future remnant liver volume
RLV: Remnant liver volume
ROC: Receiver-operating characteristic
EGFR: Epidermal growth factor receptor
BMI: Body mass index
ICG: Indocyanine green
